# Effects of cotton straw biochar on methane emissions using rumen simulations

**DOI:** 10.1371/journal.pone.0336804

**Published:** 2025-12-04

**Authors:** Shiqi Zhang, Yaqian Zhang, Yankun Zhao, Nan Zheng, He Chen

**Affiliations:** 1 Laboratory of Quality and Safety Risk Assessment for Agro-Products of Ministry of Agriculture and Rural Affairs, Key Laboratory of Agro-Products Quality and Safety of Xinjiang, Institute of Quality Standards& Testing Technology for Agro-Products, Xinjiang Academy of Agricultural Sciences, Urumqi, China; 2 State Key Laboratory of Animal Nutrition and Feeding, Institute of Animal Sciences, Chinese Academy of Agricultural Sciences, Beijing, China; University of Illinois, UNITED STATES OF AMERICA

## Abstract

**Context:**

Cotton stalk valorization is limited by high lignification, resulting in waste and environmental issues. Biochar, a pyrolysis-derived material, shows promise in reducing ruminant methane emissions. Aims: This study investigates the effects of cotton stalk biochar (CSB) as a feed additive on rumen fermentation, methane emissions, microbial communities, and nutrient degradation in dairy cows.

**Methods:**

Cotton stalks were air-dried, cut into 2–3 cm segments, oven-dried at 105°C for 6 h, ground through an 80-mesh sieve, and pyrolyzed under N_2_ (0.5 L/min) at 13.3°C/min to 400°C with a 3 h hold to produce CSB. CSB was added to diets at 0%, 3%, and 6% levels. Each treatment was conducted in triplicate, with two fermentation bottles for each replicate. Rumen fermentation was simulated using an in vitro static culture method with rumen fluid from Holstein dairy cows. Fermentation parameters, gas production, nutrient disappearance, microbial populations, and metabolites were analyzed.

**Key Results:**

CSB addition increased rumen pH and significantly reduced total gas, CH_4_, CO_2_, and H_2_ production (*P* < 0.05). Total volatile fatty acid concentration decreased with increasing CSB levels. However, no significant changes were observed in microbial diversity or total DNA copy numbers of bacteria and methanogens. Metabolomics revealed that CSB altered key metabolites involved in amino acid (such as citrulline) and fatty acid metabolism (such as pyruvic acid and undecanoic acid). As CSB levels increased, cellulase activity decreased, thereby inhibiting dry matter and fiber loss rates and further reducing gas production. Conclusions: In conclusion, the addition of 3% or 6% biochar to the feed of cows was able to minimise ruminal gas production. However, it may act by inhibiting the ruminal fermentation (NDF degradation) pathway.

**Implication.:**

The addition of 3% CSB can reduce in vitro gas production in the rumen of dairy cows.

## 1. Introduction

The high lignification of cotton stalks and immature utilization technologies lead to significant resource waste and environmental issues, making enhanced valorization crucial for sustainability. Biochar—produced via oxygen-limited biomass pyrolysis—offers a multifaceted solution, gaining attention for applications including water purification, syngas upgrading, and notably, livestock methane mitigation [1]. Its structural properties (high surface area and porosity) enable rumen microbiome modulation, improved fermentation efficiency, and reduced methane emissions [2]. Specifically, the adsorption capacity of biochar adsorbs substrates, metabolites, and methane precursors, thereby regulating the ruminal environment and inhibiting the activity of methanogenic bacteria. Meanwhile, its porous structure provides habitats for microorganisms, promotes synergistic interactions among them, and affects substrate diffusion and transport. These actions collectively improve microbial metabolic efficiency and substrate utilization rate, optimize the ruminal fermentation process, and reduce methane emissions.

Studies confirm biochar’s positive impact on ruminant productivity. Pine-derived biochar enhanced in vitro rumen fermentation, nutrient digestibility, microbial protein synthesis, and significantly reduced enteric methane by up to 20% [3]. Practical applications show that 0.6% rice husk biochar increased cattle weight by 25% and reduced 22% methane production [2], while combining rice byproducts with 1% rice husk biochar boosted weight gain by 60% and improved feed conversion ratio (FCR) [4]. Supplementing urea-molasses blocks with 2–8% rice husk biochar similarly enhanced growth by 43% and optimized FCR [5]. However, biochar’s methane-mitigation efficacy varies considerably, being highly dependent on feedstock composition and pyrolysis parameters (temperature, residence time, heating rate) [6–8]. These studies demonstrate consistent benefits at optimal inclusion levels, with methane reduction ranging from 10% to 30% depending on the biochar type and application method [4,5,9–12]. Studies using diverse materials and methods report significant fluctuations in methane reduction outcomes, highlighting the need for careful feedstock selection. While cotton stalks—rich in lignin/cellulose—are suitable for conversion into highly porous biochar via high-temperature pyrolysis, their specific mechanisms for modulating rumen metabolism and methanogenesis remain unexplored.

In this study, we hypothesized that cotton stalk biochar (CSB) synergistically reduces methane emissions by remodeling the rumen microbiota, inhibiting methanogenic activity, and optimizing fermentation efficiency. This study aimed to explore the effects of CSB on gas production, microbial community composition and nutrient degradation using in vitro rumen simulation technology, as well as to determine the optimal CSB inclusion level to balance methane mitigation and nutrient utilization, and to provide a theoretical basis for the use of CSB as a sustainable feed additive for ruminant animals.

## 2. Materials and methods

### 2.1. Ethical approval

All procedures were approved by the Animal Care and Use Committee of the Institute of Animal Sciences, Chinese Academy of Agricultural Sciences, Beijing, China (Protocol Number: IAS 2024−20).

### 2.2. Cotton straw biochar preparation

Fresh cotton stalks were air-dried, sectioned into uniform 2–3 cm segments, and oven-dried at 105 °C for 6 hours to ensure consistent pyrolysis conditions. The processed feedstock was ground through an 80-mesh sieve and loaded into a ceramic reactor. Under continuous nitrogen purging (0.5 L/min) to maintain an anoxic environment, pyrolysis was conducted with strict temperature control: heating from ambient to 400 °C at 13.3 ± 0.5 °C/min, holding at 400 °C for 3 hours, and cooling to ambient temperature under nitrogen atmosphere. The elemental composition of the resultant biochar is detailed in Table 1 and 2.

**Table 1:** Composition and nutrient level of the basal diet.

Feed ingredients1, % of DM	Content
Corn	23.00
Soybean meal(46%CP)	13.00
Alfalfa hay	15.00
Corn silage	45.00
Premix2	3.00
Urea	1.00
Total	100.00
Nutrient level, % of DM	Content
CP	16.21
EE	2.66
NDF	35.28
ADF	22.85
Ash	6.61

DM = dry matter; CP = crude protein; NDF = neutral detergent fiber; ADF = acid detergent fiber; EE = ether extract.

^1^The basal diet was formulated according to NRC (2001) for dairy cows.

^2^Concentration per kilogram of premix DM: 4 g of calcium, 5.5 g of phosphorus, 2 g of sodium, 4 g of magnesium, 40 000 IU of vitamin A, 37,000 IU of vitamin D, 500 IU of vitamin E, 30 mg of copper, 25 mg of iron, 140 mg of manganese, 140 mg of zinc, 0.8 mg of selenium.

**Table 2:** CSB elemental content.

Cotton straw biochar elemental content			
O, %	17.04	Se, mg/kg	0.1
C, %	70.78	Mo, mg/kg	1.72
N, %	1.13	Ag, mg/kg	0.04
S, %	0.31	Cd3, mg/kg	0.02
K, %	2.58	Pb2, mg/kg	0.49
Na, %	0.26	B, mg/kg	28.7
Mg, %	0.31	Si, mg/kg	160
P, %	0.25	Co, mg/kg	0.15
Al, mg/kg	185	Ni, mg/kg	0.69
Fe, mg/kg	217	Cu, mg/kg	12.3
Mn, mg/kg	19.6	As, mg/kg	0.15
Cr1, mg/kg	3.64	Hg, mg/kg	0.001
Zn, mg/kg	21.6	Specific surface area4, m2/g	18.07

1,2,3 Under the condition of CSB addition amount in this study, the indexes of heavy metals were in line with the safe use of feed in China.

4 This data is calculated using the Brunauer-Emmett-Teller equation after determining the relevant parameters.

### 2.3. Experimental design

In this experiment, batch cultivation was conducted using the anaerobic static culture method, and three-run experiments (n = 3) were carried out. Each run included three treatment methods. The content of cotton stalk biochar-based substrate feed used for each treatment was 0%, 3%, and 6% of dry matter content, and each treatment had 2 fermentation bottles. Ruminal fluid was collected on three consecutive days from three Holstein dairy cows (body weight 576 ± 28.5 kg), each with a rumen fistula, and used in three separate fermentation batches. The mixed liquids were filtered through four layers of gauze. The mixed feed formulation for fistula cows was the same as in Table 1. Feeding was done three times a day.

The composition and nutritional profile of the basal diets are detailed in Table 1. Each fermentation system was accurately weighed, and 2 g of feed was sealed in a fiber bag (pore size: 0.5 mm, China Agricultural University). The feed particles could not enter and exit the fibre bag freely, which reduced the influence on sample collection and facilitated the collection of liquid phase microbiological samples. Rumen fluid was collected in the morning before feeding. Each fermentation system was fermented in 100 mL fermentation bottles. The fermentation solution prepared according to the method outlined by reference [13] had a 1:2 ratio of filtered rumen fluid to buffer solution, totalling 60 mL. The bottles were sealed in an oxygen-free carbon dioxide environment and incubated in a shaker at 39 °C, 120 rpm/min for 24 h.

### 2.4. Sample collection and processing

Fermentation broth samples (2 mL each) were collected at 0, 3, 6, and 12 hours during fermentation. Immediately after sampling, the pH value (1 mL) was measured with a pH sensor (inpro31001/120 Pt100, Mettler Toledo Technology Co., LTD., China). The second aliquot (1 mL) was centrifuged at 13,000 × g for 10 min. At each time point, 300 µL was mixed, vortexed, and stored at −80 °C until metabolomics analysis. One portion (1 mL) was used to measure the pH value using a pH sensor (InPro3100/120 Pt100, Mettler-Toledo Technology Co., Ltd., China). The second aliquot (1 mL) was centrifuged at 13,000 × *g* for 10 minutes. At each time point, 300 µL was taken and mixed together, vortexed and mixed for metabolomic analysis. After rinsing the fermented feed with water until clear, it was dried together with the original feed in a forced air drying oven at 65 °C for 48 hours. The dried material was pulverized, dried at 105 °C, and then subjected to chemical analysis using a Cyclotec mill (Cyclotec 1093, Foss Electric, USA) with a 1-mm sieve.

### 2.5. Fermentation index determination

At 3, 6 and 12 h of fermentation, the fermentation flask air pressure was measured with a digital manometer (CPG500, WIKA, Germany) and the total gas production was calculated according to Zhang et al. [14]. All gases at this time point were collected using a gas collection bag (50 mL, Beekman Biotechnology Co., Ltd., Hunan, China). Gas composition content was analyzed using a GC112A chromatograph (Shanghai Yidian, China) with a 5A stainless steel column (Φ3mm×3m, 60–80 mesh Chromosorb) and HW-2000 workstation. Operating conditions: column, TCD, and injection port temperatures were set to 100 °C; injection volume, 1 mL; carrier gas, high-purity argon at 30 mL/min and 0.4 MPa. The CO_2_, H_2_ and CH_4_ production at different points in time is calculated according to the following formula: Gas production (mL)= Gas composition content (%) × Total gas production (mL)[15]. Volatile fatty acids (VFA) were determined by gas chromatography using 4-methyl-N-valeric acid as an internal standard [16]. Ammonical nitrogen (NH_3_-N) was determined using an alkaline sodium hypochlorite-phenol spectrophotometric method [17].

### 2.6. Nutrient determination

Nutritional composition analysis included determining nitrogen content by combustion (AOAC Method 990.03, 2016) [18] to calculate crude protein, analyzing ash content and ether extract (EE) using AOAC Method 942.05 (2016) [18], measuring neutral detergent fiber (NDF) and acid detergent fiber (ADF) according to Van Soest et al. [19] and Goering et al. [20], assessing dietary sulfur by ICP-MS (Agilent 7900), and calculating the nutrient disappearance rate as [(Initial – Residual content)/ Initial content] × 100% [21]. Protease activity was measured using a colorimetric assay kit (Nanjing Jiancheng Bioengineering Institute, Cat. No. A080-3–1), while cellulase activity was determined using another colorimetric assay kit (Boxbio, Cat. No. AKSU043C)[22].

### 2.7. Microbial community structure and populations

Total DNA was extracted from fermentation broth using CTAB and bead-beating [23]. Concentration and purity were measured by NanoDrop (Thermo Fisher Scientific, Waltham, USA). Real-time PCR quantified DNA copy numbers for various microbial species and groups using primers from Denman et al. [24] Zhou et al. [25], and Yu et al. [26] as shown in Table 3. The V3-V4 region of 16S rDNA was amplified by PCR with primers 338F (ACTCCTACGGGAGGCAGCA) and 806R (GGACTACHVGGGTWTCTAAT). The V4-V5 region of the 16S rRNA gene for archaea was amplified using specific primers 915F (CAGCCGCCGCGGTAA)and 915R (GTGCTCCCCCGCCAATTCCT). The 16S rDNA data underwent rigorous processing. Initially, data were filtered and merged using FASTP version 0.14.1 (https://github.com/OpenGene/fastp) to yield valid, contiguous segments. Subsequently, operational taxonomic units (OTUs) clustered at a 97% sequence similarity threshold using UPARSE v11, and species annotation was achieved using usearch -sintax against the SILVA (16S) database with a confidence threshold of 0.8. The raw sequence data of 16S rRNA is available in Mendeley Data (https://doi.org/10.17632/kcdw49w9ch.1).

**Table 3:** Rumen microflora gene primer information.

Genes	Primer sequences	GenBank accession No.	Annealing temperature/°C	Product length/bp
Total bacteria	F: CGGCAACGAGCGCAACCC	CP058023.1	60	147
	R: CCATTGTAGCACGTGTGTAGCC			
Total methanogens	F: TTCGGTGGATCDCARAGRGC	GQ339873.1	60	160
	R: GBARGTCGWAWCCGTAGAATCC			

LEfSe analyses were conducted to identify species exhibiting significant abundance differences between subgroups. This involved a non-parametric factorial Kruskal-Wallis sum-rank test, followed by linear discriminant analysis (LDA) for effect size estimation. An LDA score of 2 was employed as the default threshold. Additionally, R software was utilized to perform shared and endemic species statistics, community composition analysis, alpha and beta diversity assessments, and LEfSe analysis, with statistical significance declared at *P* < 0.05. Raw data analysis, including peak extraction, baseline adjustment, deconvolution, alignment, and integration, was finished with the Chroma TOF (v 4.3x, LECO) software. The LECO-Fiehn RTX-5 database was used for metabolite identification by matching the mass spectrum and retention index. Finally, the peaks detected in less than half of the QC samples or RSD > 30% in QC samples were removed.

### 2.8. Metabolomic analysis of volatile and non-volatile metabolites

#### 2.8.1. GC-MS analysis.

A 100 μL sample was mixed with 400 μL of pre-cold methanol containing internal adonitol (0.5 mg/mL) and vortexed for 30 seconds. The mixture was then subjected to ultrasonic treatment in an ice-water bath for 10 minutes, followed by centrifugation at 4°C for 15 minutes at 12000 rpm. The supernatant (400 μL) was transferred to a fresh tube and dried in a vacuum concentrator. For the quality control (QC) sample, 70 μL of each sample was pooled. After drying, 30 μL of methoxyamination hydrochloride (20 mg/mL in pyridine) was added and incubated at 80°C for 30 minutes. Derivatization was performed by adding 40 μL of BSTFA reagent (1% TMCS, v/v) and incubating at 70°C for 1.5 hours. The samples were cooled to room temperature, and 5 μL of FAMEs (in chloroform) was added to the QC sample. All samples were analyzed using GC-MS (Vanquish, Thermo Fisher Scientific).

#### 2.8.2. Nuclear magnetic resonance analysis.

Non-volatile metabolomic profiling was conducted using a UPLC-HRMS platform. Automated sample preparation was performed on ice, where 100 μL aliquots were extracted with 400 μL ice-cold methanol: acetonitrile (1:1 v/v) containing a commercial isotope-labeled metabolite mix for quality control. After vortexing, incubation, and filtration, supernatants were analyzed on a Vanquish UPLC-Orbitrap Exploris 120 system equipped with a BEH Amide column (Vanquish, Thermo Fisher Scientific). The chromatographic gradient was programmed from 95% B (acetonitrile) to 50% B over 1–15 min, then returned to 95% B within 1 min, and held for 4 min equilibration (total run: 20 min) at 0.3 mL/min flow. High-resolution mass spectrometry operated in polarity-switching mode with stepped collision energies, achieving 60,000 full-scan and 15,000 MS/MS resolution. System stability was monitored via QC samples injected every 10 runs.

Raw data were processed through ProteoWizard (v3.0) conversion and XCMS-based (v3.12) feature detection (m/z window 0.015, retention time bandwidth 5 min). Stringent quality filters removed features with >30% missing values in QCs or QC RSD > 25%, followed by probabilistic quotient normalization. Metabolite identification required: (1) mass accuracy <3 ppm versus BiotreeDB (V3.0); (2) MS/MS spectral match (dot product >0.7); (3) retention time deviation <0.2 min for authenticated standards. Multivariate analysis (PCA/PLS-DA) of 36,165 detected features from 20 biological replicates was implemented using custom R scripts, adhering to recent metabolomics reporting guidelines [27]. Rumen metabolomic datasets underwent PCA and PLS-DA within the MetaboAnalyst 6.0 platform [28]. The PLS-DA model identified discriminant features using a variable importance in projection (VIP) score ≥2, and metabolic pathway enrichment was deemed significant at *P* < 0.05.

### 2.9. Statistical analysis

In vitro gas production data were analyzed using SAS 9.2 (SAS Inst. Inc., Cary, NC, USA), employing the MIXED model to evaluate differences across various time points and treatment groups. Time and treatment were considered fixed effects, and a Tukey-Kramer test was applied for multiple comparisons. The *in vitro* fermentation data were calculated from the following model:


Yijkl=μ+Trtk+Runi:k+Timel+Trt×Timekl+eijkl

In this model, *Yijkl* was the dependent variable, *μ* was the least squares mean, Trt*k* was the fixed effect of the *k*th treatment (*k* = 0, 3%, 6%). Run*i:k* was the random effect of the *i*th run (*i* = 1, 2, 3) in the *k*th diet treatment. Time*l* was the effect of repeated measurements at time *l* (*l* = 0, 3, 6, 12, 24 h), Trt × Time*kl* was the interaction effect between the *k*th diet treatment and time *l*, The *eijkl* is the random error associated with ijkth data value assuming that *eijkl* is independently identically N (0, σ2). A Tukey-Kramer test was used for multiple comparisons of differences.

For DNA copy number, NH_3_-N, VFA, nutritional disappearance and digestive enzyme activity (cellulase and protease) data analyses, an ANOVA model was used, followed by Duncan’s multiple range test. The outcomes were presented as least square means along with their standard errors (SE), with statistical significance set at *P* < 0.05.

## 3. Results

### 3.1. Rumen fermentation parameters

As the CSB additive level rises, the pH increases, with 3% and 6% groups significantly higher than the 0% group (*P* < 0.05, Table 4). The total gas, methane, CO_2_ and hydrogen production all decrease, with 3% and 6% groups significantly lower than the 0% group (*P* < 0.05). Specifically, total gas production decreased by 6.4% in the 3% group and by 12.9% in the 6% group. CH_4_ production decreased by 7.9% in the 3% group and by 10.9% in the 6% group. CO_2_ production decreased by 3.8% in the 3% group and by 14.3% in the 6% group. H_2_ production decreased by 20.0% in the 3% group and by 20.0% in the 6% group. NH_3_-N concentration showed no significant differences among treatment groups, ranging from 25.26 to 25.46 mg/dL (*P* = 0.089). The total volatile fatty acid concentration declined as the CSB level increased (*P* < 0.05), with the 6% group being significantly lower than the 0% group in total volatile fatty acids, acetic acid, propionic acid, butyric acid, and isovaleric acid (*P* < 0.05). Butyrate, isobutyrate, valerate, and the acetate-to-propionate ratio showed no significant differences among the groups (*P > *0.05).

**Table 4:** Rumen fermentation parameters.

Item	Cotton straw biochar levels			SE1	*P*-value		
	0%	3%	6%		Treatment	Time	Interaction
pH value	6.75a	6.81b	6.83c	0.054	<0.001	<0.001	0.384
Total gas production, mL	77.88a	72.89b	67.86c	10.225	<0.001	<0.001	0.474
CH_4_ production, mL	4.03a	3.71b	3.59c	0.584	<0.001	<0.001	0.920
CO_2_ production, mL	37.06a	35.66b	31.75c	4.961	<0.001	<0.001	0.265
H_2_ production, mL	0.05a	0.04b	0.04b	0.007	<0.001	<0.001	0.760
NH_3_-N, mg/dL	25.26	24.27	25.46	0.376	0.089	/	/
Total Volatile fatty acids, mmol·L-1	79.81a	77.54a	69.89b	2.872	0.009	/	/
Acetate, %	63.28a	62.50ab	60.59b	0.898	<0.001	/	/
Propionate, %	23.40a	23.83a	25.52b	0.642	<0.001	/	/
Butyrate, %	10.88	11.35	11.56	0.268	0.237	/	/
Isobutyrate, %	0.69a	0.64b	0.65ab	0.019	<0.001	/	/
Valerate, %	1.09	1.06	1.10	0.045	0.782	/	/
Isovalerate, %	0.64a	0.60ab	0.58b	0.022	<0.001	/	/
Acetate: Propionate	2.71	2.66	2.44	0.174	0.291	/	/

^1^SE = standard error of least square means.

^a-c^Least square means values within a row with different superscripts differed (*P* < 0.05)

### 3.2. Nutritional disappearance and digestive enzyme activity

The dry matter disappearance rate was significantly lower in the 6% biochar group compared to both the control and 3% biochar groups (*P* < 0.001, Table 5), with no statistical difference observed between the 3% and 0% treatment. Similarly, the crude protein disappearance rate exhibited a marked reduction in the 6% biochar group (*P* < 0.001), whereas the 3% biochar treatment showed comparable degradation efficiency to the 0% treatment. For neutral detergent fiber degradation, the 6% biochar group demonstrated significantly impaired fiber breakdown (*P* < 0.001), while the 3% biochar addition did not differ significantly from the 0% in this parameter. Cellulase activity decreased significantly with increasing biochar supplementation levels (*P* = 0.004). The 6% biochar group (2.47 ± 0.193 U/mL) exhibited a 32.1% decline compared to the control group (3.64 ± 0.193 U/mL). In contrast, protease activity showed no significant alterations (range: 19.83–22.23 U/mL; *P* = 0.325). These findings collectively indicate a dose-dependent inhibitory effect of biochar supplementation on diet degradation at higher inclusion levels.

**Table 5:** Feed nutrient disappearance rate.

Item	Cotton straw biochar levels			SE1	*P*-value
	0%	3%	6%		
Dry matter disappearance rate, %	59.77a	59.33a	56.38b	0.328	<0.001
Crude protein disappearance rate, %	53.27	53.23	52.92	1.262	0.978
Neutral detergent fiber disappearance rate, %	44.22a	42.50a	36.82b	0.553	<0.001
Acid detergent fiber disappearance rate, %	46.89a	45.98a	41.31b	0.648	<0.001
Protease, U/mL	19.83	20.35	22.23	1.179	0.325
Cellulase, U/mL	3.64	3.06	2.47	0.193	0.004

^1^SE = standard error of least square means.

^a-b^Least square means values within a row with different superscripts differed (*P* < 0.05)

### 3.3. Microbial population and microbial community composition

**Fig 1:**
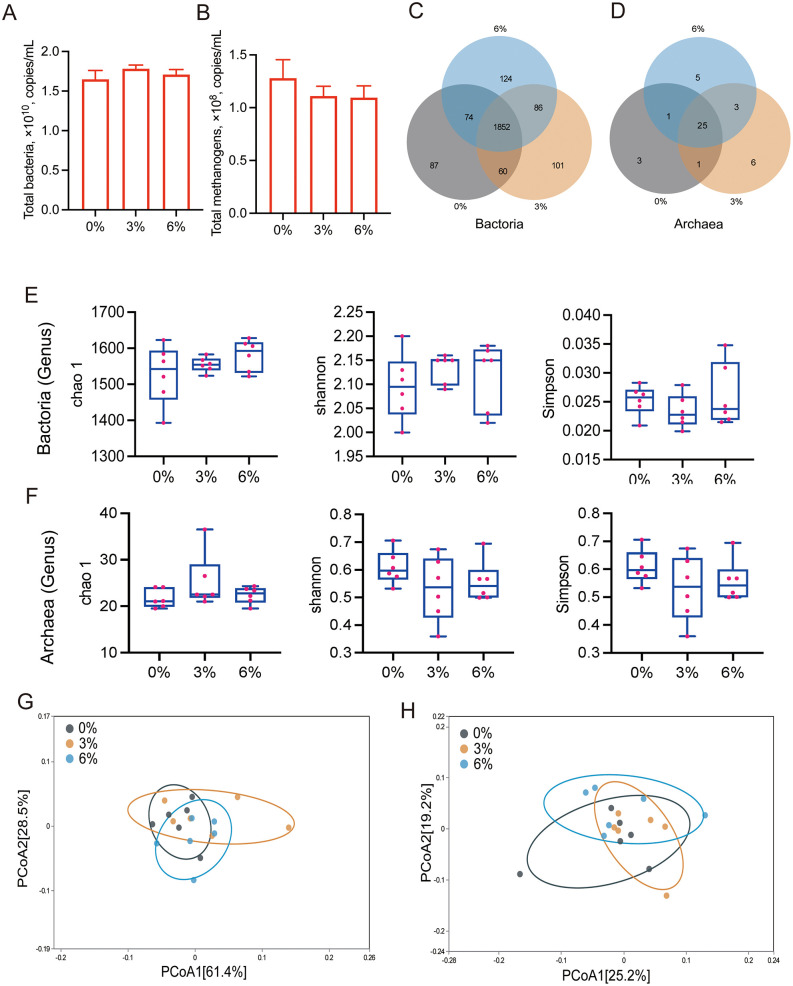
Rumen bacterial and archaeal diversity. (A) Total bacterial DNA copies. (B) Total methanogen DNA copies. (C) Bacterial Venn diagram. (D) Archaeal Venn diagram. (E) Bacterial alpha-diversity indices. (F) Archaeal alpha-diversity indices. (G) Bacterial beta-diversity indices. (H) Archaeal beta-diversity indices.

The addition of cotton straw biochar did not significantly affect the total bacterial DNA copy number in the rumen (Fig 1A) or the total methanogen DNA copy number (Fig 1B; *P* > 0.05). The 0%, 3%, and 6% treatment groups showed bacterial OTU numbers of 2,073, 2,099, and 2,136, respectively, while archaeal OTU numbers were 30, 34, and 35 in the corresponding groups (Fig 1C, D; *P* > 0.05). No significant differences were observed in alpha diversity (Fig 1E, F) or beta diversity (Fig 1G, H) of bacteria and archaea among the groups.

### 3.4. Fermentation metabolites

**Fig 2:**
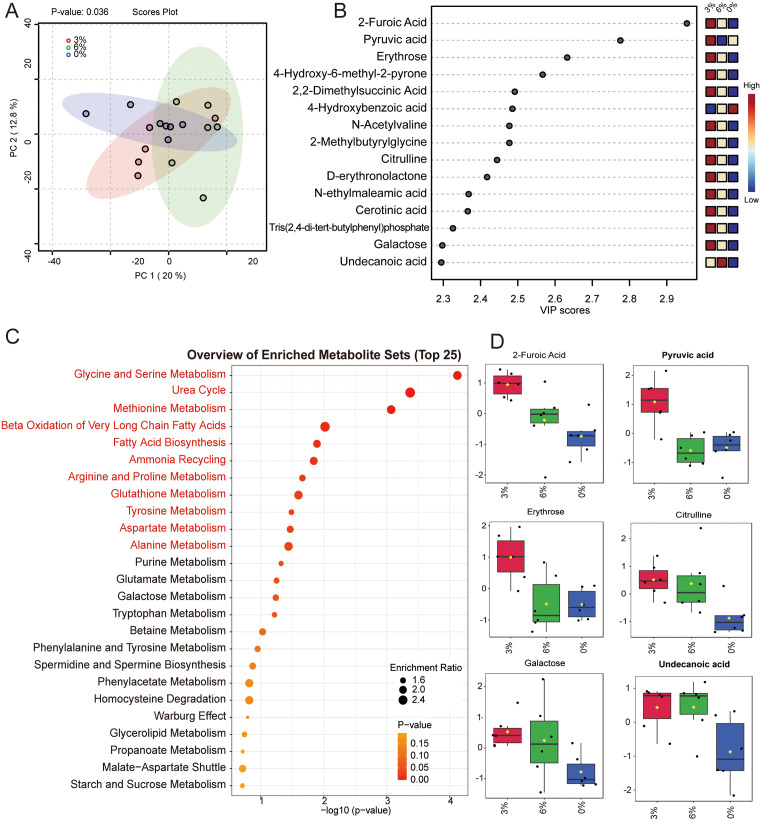
Changes in rumen fluid metabolites. (A) PCA score plot. (B) PLS-DA score plot. (C) KEGG bubble plot. (D) Key differential metabolites.PCA and PLS-DA plots display metabolite distribution differences among 0%, 3%, and 6% CSB groups, with axes representing principal components/latent variables and points colored by group (red = 0%, blue = 3%, green = 6%). The KEGG plot illustrates enriched metabolic pathways, with bubble size indicating metabolite count and color showing significance (-log10 P-value; red = significant). The differential metabolites plot highlights key metabolites by VIP score, with higher scores indicating greater importance.

A total of 302 volatile metabolites and 1,470 non-volatile compounds were detected in this study. Principal component analysis revealed a partial separation trend among the sample groups along the dimensions of PC1 (32.1%) and PC2 (18.7%) (*P* = 0.036), indicating significant differences in their metabolic profiles. This suggests that CSB regulates the metabolic networks within the rumen (Fig 2A). Through the projection of variable importance (VIP > 2) combined with t-tests (*P* < 0.05), key metabolite in 3% group s such as 2-Furoic Acid, Pyruvic acid, Erythrose, Citrulline, Cerotinic acid, Galactose, and Undecanoic acid were identified (Fig 2B, D). These metabolites significantly regulate metabolic pathways in the rumen, including amino acid metabolism, fatty acid metabolism, and the urea cycle (*P* < 0.05).

## 4. Discussion

This study aimed to explore the potential of CSB in modulating rumen metabolism and methane emissions by supplementing dairy cow diets with 3%–6% CSB and investigating its effects on in vitro ruminal fermentation. Contrary to our hypothesis, the results of the present study demonstrated that although CSB supplementation significantly reduced fermentative gas production, it also markedly affected rumen fermentation function, including decreases in total VFA, dry matter disappearance, NDF disappearance rate, and cellulase activity. These changes suggest that CSB may reduce methane production primarily by decreasing cellulose digestibility and reducing available substrates, rather than through direct inhibition of methanogen activity.

Specifically, the 3% CSB supplementation enhanced amino acid and fatty acid metabolism to some extent but did not significantly affect nutrient digestibility. However, when the supplementation level was increased to 6%, fermentation function was significantly inhibited, as evidenced by notable declines in dry matter disappearance rate, NDF disappearance rate, and cellulase activity. This indicates that higher doses of CSB may adversely affect animal digestive function, consistent with previous observations in diets containing high levels of rice husk (>5%) [29]. The impact of biochar as a feed additive on methane emissions during rumen fermentation varies considerably across studies. Several reports indicate that supplementing grass hay or barley silage-based diets with biochar from different sources and at varying concentrations did not significantly reduce methane production [30]. However, some experiments have shown that adding 1% biochar can reduce in vitro ruminal methane flux by 12%–49% [2], and synergistic effects with bio-fats or sucrose loading treatment also contribute to methane mitigation [31,32]. In contrast, potato peel biochar and agroforestry-derived biochar at 10% inclusion significantly increased methane production [33]. These discrepancies highlight that the influence of biochar on methane emissions is comprehensively affected by multiple factors, including its source, inclusion level, processing methods, and feed composition, indicating a complex mechanism of action. Future research should further investigate the interactions among these factors to optimize biochar application and develop more effective methane mitigation strategies, thereby promoting sustainable dairy production.

The reduced dry matter disappearance rate observed in this study suggests that CSB supplementation decreased feed digestibility. The declines in NDF degradation and VFA production further support the negative impact of CSB on cellulose degradation. The reduction in cellulase activity indicates that CSB may impair cellulose degradation by inhibiting enzymatic activity, potentially due to the physical adsorption of nutrients, enzymes, and microbes onto the high specific surface area of CSB [34]. (1) The well-developed pore structure (particularly micropores) of high-surface-area biochar enables strong adsorption of nutrients such as nitrogen and phosphorus, limiting microbial access to these substrates, suppressing metabolic activity, and consequently impairing cellulose degradation [35]. (2) Biochar surfaces can adsorb cellulases (e.g., β-glucosidase) via hydrophobic interactions, hydrogen bonding, or electrostatic forces, leading to enzyme inactivation. Experiments by Foster et al. [35] demonstrated that β-glucosidase adsorbed onto biochar lost over 95% of its activity, primarily because enzyme molecules were immobilized within pores and unable to access substrates. Additionally, as noted in [36], surface functional groups (e.g., aromatic structures) on biochar can adsorb enzyme proteins via π–π interactions, further contributing to reduced cellulose degradation.

However, biochar did not affect crude protein disappearance rate or protease activity, suggesting selective adsorption toward cellulases or their substrates rather than proteases. Proteases generally possess a more compact globular structure, whereas cellulases are large multidomain molecules (e.g., containing carbohydrate-binding modules). Research by Foster et al. [35] showed that biochar adsorbed β-glucosidase (a large molecule) more strongly than smaller molecules like phosphatase, implying that proteases may be less susceptible to micropore capture due to their smaller size or structural properties. Moreover, biochar may preferentially adsorb negatively charged cellulases (with hydrogen bonding dominating adsorption at low pH), while proteases, which may carry different surface charges (e.g., positive) near neutral pH, could exhibit weaker competitive adsorption [37]. Studies by An et al. [38] indicated that biochar adsorption of Pb2 + increased at pH > 6, suggesting that its negatively charged surface under alkaline conditions favors cation adsorption (e.g., metal cofactors). However, proteases generally rely less on metal ions.

The 6% level significantly reduced digestibility and fermentation efficiency, raising concerns about potential negative impacts on animal productivity. Therefore, future research should focus on the following key areas: First, modifying the biochar surface to reduce non-target adsorption of nutrients, enzymes, and microbes, thereby mitigating the negative effects on cellulose degradation. Optimizing preparation processes to adjust the physical and chemical properties of biochar may also help minimize adverse impacts on rumen fermentation [39]. Second, investigating synergistic effects between CSB and other additives (e.g., propionate promoters, probiotics, enzymes) to improve rumen fermentation efficiency and enhance methane mitigation [40]. Third, conducting long-term in vivo trials to comprehensively evaluate the effects of CSB on animal health (including growth performance, reproductive capacity, and immune function) and the environment (e.g., soil quality, water quality, and ecosystem health)[41]. Furthermore, further optimization of CSB inclusion levels is necessary to identify a dosage that effectively reduces methane emissions without significantly compromising animal productivity. Studies on the responses of different animal breeds to CSB should also be conducted to develop more targeted feed formulations. Finally, the economic feasibility of CSB as a feed additive should be assessed, including its production costs, transportation expenses, and impacts on animal performance, along with investigations into its market potential and application prospects to facilitate widespread use in sustainable ruminant production.

## 5. Conclusions

In conclusion, under the conditions of this experiment, addition of 3% or 6% biochar appeared to reduce ruminal fermentation and gas production by inhibiting cellulase activity and thereby reducing NDF degradation. The addition of 3% CSB could reduce gas production and promote amino acid metabolism and fatty acid synthesis without affecting nutrient digestion and microbial community stability.

Future studies can focus on biochar surface modification, optimization of the preparation process, exploration of synergistic effects, long-term in vivo verification, and optimization of biochar addition amount to optimize methane reduction effect and ensure that animal performance is not significantly affected.

## Supporting information

S1 File Supplementary Materials Table 1. Feed ingredients and nutrient levels of experimental diets (%). Supplementary Materials Table 2. Rumen fermentation parameters. Supplementary Materials Table 3. Feed nutrient disappearance rate. Supplementary Materials Table 4. Volatile and non-volatile metabolomics data.
(ZIP)
